# Production of Recombinant Active Human TGFβ1 in *Nicotiana benthamiana*

**DOI:** 10.3389/fpls.2022.922694

**Published:** 2022-05-31

**Authors:** Aditya Prakash Soni, Juhee Lee, Kunyoo Shin, Hisashi Koiwa, Inhwan Hwang

**Affiliations:** ^1^Department of Life Science, Pohang University of Science and Technology, Pohang, South Korea; ^2^Department of Biological Sciences, College of Natural Science, Seoul National University, Seoul, South Korea; ^3^Molecular and Environmental Plant Sciences, Texas A&M University, College Station, TX, United States; ^4^Vegetable and Fruit Development Center, Department of Horticultural Sciences, Texas A&M University, College Station, TX, United States

**Keywords:** recombinant proteins, human growth factors, LAP-TGFβ1, human TGFβ1, *Nicotiana benthamiana*, CBM3, acid activation

## Abstract

The production of recombinant proteins in plant systems is receiving wider attention. Indeed, various plant-produced pharmaceuticals have been shown to be biologically active. However, the production of human growth factors and cytokines in heterologous systems is still challenging because they often act as complex forms, such as homo- or hetero-dimers, and their production is tightly regulated *in vivo*. In this study, we demonstrated that the mature form of human TGFβ1 produced and purified from *Nicotiana benthamiana* shows biological activity in animal cells. To produce the mature form of TGFβ1, various recombinant genes containing the mature form of TGFβ1 were generated and produced in *N*. *benthamiana*. Of these, a recombinant construct, *BiP:M:CBM3:LAP[C33S]:EK:TGF*β*1*, was expressed at a high level in *N*. *benthamiana*. Recombinant proteins were one-step purified using cellulose-binding module 3 (CBM3) as an affinity tag and microcrystalline cellulose (MCC) beads as a matrix. The TGFβ1 recombinant protein bound on MCC beads was proteolytically processed with enterokinase to separate mature TGFβ1. The mature TGFβ1 still associated with Latency Associated Protein, [LAP(C33S)] that had been immobilized on MCC beads was released by HCl treatment. Purified TGFβ1 activated TGFβ1-mediated signaling in the A549 cell line, thereby inducing phosphorylation of SMAD-2, the expression of *ZEB-2* and *SNAIL1*, and the formation of a filopodia-like structure. Based on these results, we propose that active mature TGFβ1, one of the most challenging growth factors to produce in heterologous systems, can be produced from plants at a high degree of purity *via* a few steps.

## Introduction

Plants have gained significant attention as hosts for recombinant protein production systems, with potential advantages, such as low maintenance cost, easy scalability, and no human pathogen contamination (Schillberg et al., 2003; Holtz et al., 2015). Various technologies have been developed to realize the potential of plants in the production of recombinant proteins. Of these, the most crucial is the expression vector. Various types of expression vectors have been developed that can give up to 800 mg/kg fresh weight (Maclean et al., 2007) when used in transient expression in *Nicotiana benthamiana* (Regnard et al., 2010; Werner et al., 2011). Additionally, various domains that contribute to the increase in gene expression and/or protein translation have been identified, including the M domain (Kang et al., 2018), matrix attachment regions (MARs) (Zhao et al., 2017), and various 5′ and 3′ untranslated regions (Kim Y. et al., 2014; Diamos et al., 2016).

Various recombinant proteins have been produced in plants. These include hepatitis B surface antigen (Mason et al., 1992), hemagglutinin (D’Aoust et al., 2008), consensus domain III of dengue virus E glycoprotein, cEDIII (Kim et al., 2015), and Zika virus antibodies, c2A10G6 (Diamos et al., 2020), CHKV mab (Hurtado et al., 2019), human epidermal growth factor (Wirth et al., 2004), human basic fibroblast growth factor (An et al., 2013), human growth hormone (Xu et al., 2010), human FGF (Wang et al., 2015), human interleukin 6 (Islam et al., 2018b), and E2 protein of classical swine fever virus as a vaccine (Sohn et al., 2018; Park et al., 2020).

Transforming growth factor beta (TGFβ) is a signaling molecule with crucial roles during early development and the regulation of immune responses in mammals (Wu and Hill, 2009). In animal cells, TGFβ1 is translated as a pre-pro-form that undergoes multiple proteolytic processing to produce active mature TGFβ1. During the processing of TGFβ1 to the active mature form, post-translational modification is one of the most crucial requirements to attain proper folding and trafficking to the plasma membrane. The pro-form of TGFβ1, LAPTGFβ1, is glycosylated at three residues (the amino acid positions of 82, 136, and 176). Among these, Asn82 and Asn136 are necessary for the proper secretion of LAPTGFβ1 (Sha et al., 1989; Brunner et al., 1992). Studies on the molecular events in the processing of the precursor showed that proteolytic processing occurs twice to produce the mature form. After synthesis, the first proteolytic cleavage occurs between Gly29 and Leu30 of pre-pro-TGFβ1, thereby yielding pro-TGFβ1 (amino acids 30 to 390). Proteolytic processing of the pro-TGFβ1 occurs at a cluster of basic amino acid residues (R-H-R-R) immediately preceding Ala279 to yield the mature TGFβ1. The processing site conforms to a consensus cleavage motif for the mammalian convertase furin. Despite the cleavage, TGFβ1 dimers remain attached to the LAP domain non-covalently even after secretion and only get released upon activation (Khalil, 1999). This tight regulation prevents undesired interaction with ubiquitous receptors at the cell surface. These complicated processing steps render the production of active TGFβ1 as a recombinant protein highly difficult. In the recombinant TGFβ1 protein production system without Latent TGFβ1-binding protein (LTBP), C33 has the capability to form an intramolecular disulfide bond with a cysteine residue in mature TGFβ1 (Gentry et al., 1987), which then inhibits the release of mature TGFβ1 dimers from the LAP without activation. When the C33S mutation was introduced into LAP mature TGFβ1 was more easily released from LAP-TGFβ1 (pro-TGFβ1) (Brunner et al., 1992).

In this study, we investigated whether we can produce and purify transforming growth factor-beta 1 (TGFβ1) in *N. benthamiana*. TGFβ1 also plays a very important role in wound healing, one of the most complicated processes that require complex coordination among cells, starting with the influx of inflammatory cells, epithelial to mesenchymal cells trans-differentiation, and extracellular matrix formation. The topical application of TGFβ has been shown to improve healing (Clark, 1996). In a canine model, TGFβ1 and TGFβ2 have been shown to enhance bone formation (Ruskin et al., 2000; Sumner et al., 2001). Because of its clinical importance, there is a high demand for recombinant TGFβ1. Indeed, in *E*. *coli*, the mature form of TGFβ1 was produced as an inclusion body. Subsequently, active TGFβ1 was produced from the inclusion bodies by several cycles of denaturation and renaturation steps (Kim Y.V. et al., 2014). Recently, active human TGFβ1 has been successfully produced and purified from CHO cells at a yield of 30 mg per liter (Zou and Sun, 2004). In addition, an attempt was made to produce TGFβ1 in *N*. *benthamiana*. The Latency Associated Protein (LAP) domain together with the mature TGFβ1, LAPTGFβ1, was expressed and processed into a biologically active form through in-planta cleavage by co-expression of protease, *furin*, a convertase (Wilbers et al., 2016). However, in this case, the expression level was too low to purify active TGFβ1, although the acid-treated total soluble protein showed a certain degree of activity.

Here, to produce active mature TGFβ1, we designed a recombinant gene consisting of various domains, such as the LAP domain of TGFβ1 for proper folding and dimerization (Gray and Mason, 1990), the CBM3 domain for protein purification, and the M domain to increase the expression level. Moreover, we showed that mature TGFβ1 can be released from full-length recombinant TGFβ1 *via* proteolytic processing and activated by HCl treatment. Finally, we demonstrated that plant-produced mature TGFβ1 can activate TGFβ1-mediated signaling in animal cells.

## Results

### Design of the TGFβ1 Recombinant Gene Construct and Its Expression in *Nicotiana benthamiana*

**FIGURE 1:**
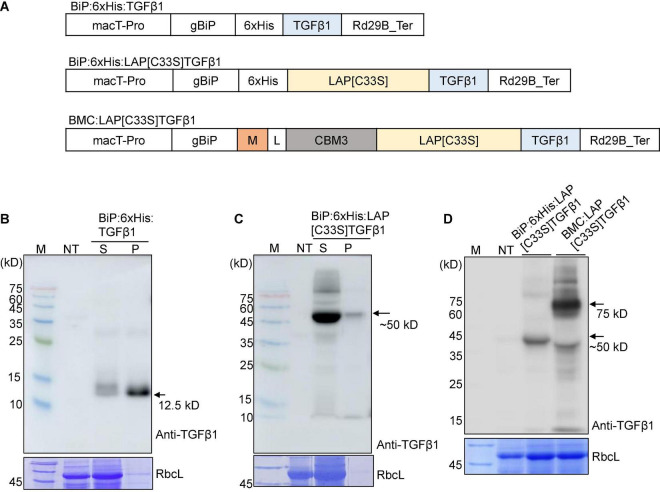
Design and expression of various *TGF*β*1* recombinant constructs to produce soluble proteins at high levels in *Nicotiana benthamiana*. **(A)** Schematic presentation of the constructs. LAP[C33S]TGFβ1, pro-TGFβ1; gBiP, genomic DNA fragment encoding the ER targeting signal (aa residues from 1 to 23) of Arabidopsis BiP1 (BAA13947); macT-pro and Rd29B_Ter, the promoter and terminator of Arabidopsis *Rd29B*, respectively; 6xHis, His tag; M, a highly N-glycosylated region (amino acid positions 231–290) of human protein tyrosine phosphatase, receptor type C (CD45); CBM3, cellulose-binding module 3 from *Clostridium thermocellum* cellulosomal scaffoldin subunit A; L, linker sequence (GGGGSGGGGS). **(B–D)** Western blot analysis of TGFβ1 recombinant proteins. The indicated constructs were transiently expressed in *N*. *benthamiana* by *Agrobacterium*-mediated infiltration. Total protein extracts from *N*. *bethamiana* leaf tissues were separated by SDS/PAGE and analyzed by Western blotting using an anti-TGFβ1 antibody. The gel was stained with Coomassie brilliant blue (CBB) to obtain the large subunit of the Rubisco complex (RbcL) band, which was used as a loading control. M, molecular weight standard; NT, non-transformed wild-type; S, total soluble protein; P, insoluble pellet fraction; arrow, recombinant protein.

To produce recombinant TGFβ1 in plants, we first determined whether the mature form of TGFβ1 could be expressed in a soluble form. In *E*. *coli*, the mature form of TGFβ1 is expressed as an insoluble inclusion body (Tuan et al., 1996; Kim Y.V. et al., 2014). Active TGFβ1 was produced from the inclusion body *via* complicated processes involving solubilization using 8 M urea and refolding using the glutathione redox system. We generated an ER-targeted N-terminal His-tagged mature TGFβ1 construct by including BiP leader sequence from Arabidopsis BiP1 giving rise to *BiP:6xHis:TGF*β*1* (Figure 1A) and expressed it in *N*. *benthamiana* using *Agrobacterium*-mediated infiltration. Total soluble protein extracts and insoluble pellet fractions were prepared from infiltrated leaves at 5 DPI and analyzed by SDS/PAGE and Western blotting using an anti-TGFβ1 antibody. BiP:6xHis:TGFβ1 (12.5 kD) was largely detected in the pellet fraction, with a minor proportion in the soluble protein extracts (Figure 1B), indicating that mature TGFβ1 is produced as insoluble aggregates in plants, as reported in *E*. *coli*. To improve the solubility of mature TGFβ1 in plants, we generated another recombinant construct of TGFβ1, *BiP:6xHis:LAP[C33S]TGF*β*1*, by including the Latency Associated Protein (LAP) domain of TGFβ1. It is known that LAP-TGFβ1 is hydrophilic, whereas mature TGFβ1 is extremely hydrophobic. In addition, we introduced the C33S mutation in the LAP domain, since the mutation causes an increase in the expression level and also makes it easy to release TGFβ1 in animal cells (Zou and Sun, 2004).

*BiP:6xHis:LAP[C33S]TGFβ1* was transiently expressed in the ER of leaf cells in *N*. *benthamiana*. Total soluble protein extracts and insoluble pellet fractions were analyzed by SDS/PAGE and Western blotting using the anti-TGFβ1 antibody. BiP:6xHis:LAP[C33S]TGFβ1 was largely present in the soluble fraction (Figure 1C), indicating that LAP[C33S] increases the solubility of the recombinant TGFβ1 protein.

The results showing that BiP:6xHis:LAP[C33S]TGFβ1 could be expressed as soluble protein in *N*. *benthamiana* prompted us to further modify the TGFβ1 recombinant gene in two directions: one to improve the expression level of the recombinant *TGF*β*1* gene and the other to incorporate an affinity tag for purification of the recombinant proteins. We explored the M domain to improve the expression level and the CBM3 domain, a cellulose binding domain, as an affinity tag for purification. Many CBDs have been identified from different fungal and bacterial proteins (Ong et al., 1989; Linder and Teeri, 1996). The M domain is a fragment (231 to 290 aa positions) of a human protein tyrosine phosphatase, receptor type C (CD45). It contains multiple N-glycosylation sites and, has been shown to enhance protein expression levels up to sevenfold when fused to a target protein (Kang et al., 2018). We fused the M and CBM3 domains sequentially to the C-terminus of the BiP leader sequence, followed by *LAP[C33S]TGF*β*1* to yield *BiP:M:CBM3:LAP[C33S]TGF*β*1* (called *BMC:LAP[C33S]TGF*β*1* hereafter). The M domain is used to increase the expression level of fusion proteins (Kang et al., 2018). Initial attempts for Ni^2+^-NTA-based purification were not successful in terms of recovery and purity of the recombinant protein. Thus, the CBM3 tag was selected as an affinity tag because it specifically binds to MCC beads with high affinity (Hong et al., 2007; Islam et al., 2018b). We expressed *BMC:LAP[C33S]TGF*β*1* in *N*. *benthamiana via Agrobacterium*-mediated infiltration, and its expression was analyzed by SDS/PAGE and Western blotting using anti-TGFβ1. The expression level of M domain-containing *BMC:LAP[C33S]TGF*β*1* was higher than that of *BiP:6xHis:LAP[C33S]TGF*β*1* (Figure 1D). A band at 50 kDa corresponded to BiP:6xHis:LAP[C33S]TGFβ1 full-length protein while in the case of BMC:LAP[C33S]TGFβ1, the full length appeared to be subjected to proteolysis and the 50 kDa band was produced that contained the C-terminal LAP[C33S]TGFβ1 region.

### TGFβ1 Recombinant Proteins Can Be Purified Using Microcrystalline Cellulose Beads

Next, TGFβ1 recombinant proteins were purified. To do this, we tested whether BMC:LAP[C33S]TGFβ1 can be purified using microcrystalline cellulose (MCC) beads, a cheap and natural resource that has been used for the purification of recombinant proteins in previous studies (Islam et al., 2018b). CBM3 strongly binds to MCC beads (Kumari et al., 2020), whereas no proteins from *N*. *benthamiana* leaf tissues were detected in the MCC bead-bound fraction. Thus, MCC beads can be used to purify CBM3-containing recombinant proteins expressed in *N*. *benthamiana*. However, CBM3-containing MCS-hIL6 cannot be eluted (Islam et al., 2018b). Thus, the target proteins can be released by proteolytic processing of recombinant proteins bound to MCC beads. In fact, in the case of TGFβ1, furin-mediated proteolysis is responsible for the release of mature TGFβ1 from the LAP domain.

**FIGURE 2:**
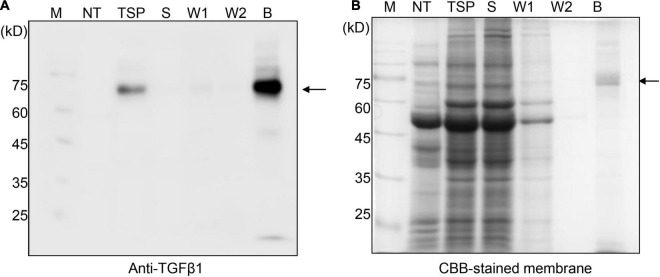
BMC:LAP[C33S]TGFβ1 shows strong and specific binding to MCC beads. **(A,B)** SDS/PAGE and Western blot analyses. Total soluble protein (TSP) extracts were incubated with MCC beads for 2 h at 4^°^C. The supernatant was collected as the unbound fraction (S) after centrifugation. The pellet containing MCC beads was collected separately and washed four times (W1–W4; only W1 and W2 were used for analysis). Proteins bound to the MCC beads were released by boiling them in SDS sample buffer. Protein samples were separated by SDS/PAGE and analyzed by Western blotting using anti-TGFβ1 antibodies **(A)** or CBB staining of the gel **(B)**. M, molecular weight standard; NT, non-transformed wild-type *N*. *benthamiana*; B, proteins bound to MCC beads; Arrow, BMC:LAP[C33S]TGFβ1.

To purify TGFβ1 recombinant proteins using MCC beads, total protein extracts were prepared from *N*. *benthamiana* leaf tissues harvested at 7 DPI and mixed with MCC beads. The MCC beads were washed extensively to remove loosely bound non-specific proteins. To analyze the purification of TGFβ1 recombinant proteins, proteins were released from MCC beads by boiling. The released proteins were analyzed by SDS/PAGE and Western blotting using an anti-TGFβ1 antibody. BMC:LAP[C33S]TGFβ1 was purified to a high degree (Figure 2) and detected as a single band by anti-TGFβ1 in both the total soluble extracts and the MCC-bound fraction. Anti-TGFβ1 did not detect any band in the unbound and washing fractions, indicating that BMC:LAP[C33S]TGFβ1 strongly binds to MCC beads.

### Substitution of Furin With Enterokinase Leads to Efficient Processing of TGFβ1 Recombinant Protein for the Release of Mature TGFβ1

Next, we examined whether mature TGFβ1 could be released from the full-length BMC:LAP[C33S]TGFβ1 recombinant protein. We used the convertase furin, which is responsible for the processing of mature TGFβ1 from the LAP domain (Dubois et al., 2001). A furin cleavage site consisting of RHRR residues is located between LAP and TGFβ1 in the native sequence. To enhance furin cleavage efficiency, we explored a different furin cleavage site by changing the sequence of the cleavage site from RHRR to RERRRKKR to yield *BMC:LAP[C33S]:F2:TGF*β*1*. To test the cleavage of TGFβ1 recombinant proteins by furin, we generated an ER-targeted furin construct, *BiP:furin_26–595_:6xHis:HDEL*. The *furin* construct was co-expressed together with *BMC:LAP[C33S]TGF*β*1* or *BMC:LAP[C33S]:F2:TGF*β*1* in *N*. *benthamiana*. To examine the release of mature TGFβ1 from BMC:LAP[C33S]TGFβ1 and BMC:LAP[C33S]:F2:TGFβ1 by BiP:furin_26–595_:6xHis:HDEL, total protein extracts from *N*. *benthamiana* leaf tissues at 3, 5, and 7 DPI were separated by SDS/PAGE and analyzed by Western blotting using the anti-TGFβ1 antibody. Even in the presence of BiP:furin_26–595_:6xHis:HDEL, the TGFβ1 recombinant protein was largely intact, as in the case without co-expression of *BiP:furin_26–595_*:*6xHis:HDEL* (Supplementary Figure 1), indicating that furin does not efficiently process both of the TGFβ1 recombinant proteins in *N*. *benthamiana*. The expression of *BiP:furin_26–595_*:*6xHis:HDEL* was confirmed by Western blot analysis using an anti-His antibody. A possible explanation might be improper or partial activation of furin. In mammalian cells, furin undergoes two-step cleavage, first in the ER and another at the trans-Golgi network. In our current study, furin was localized to the ER by using the ER retention motif HDEL, which might have caused a problem in the activation of furin in *N. benthamiana*.

**FIGURE 3:**
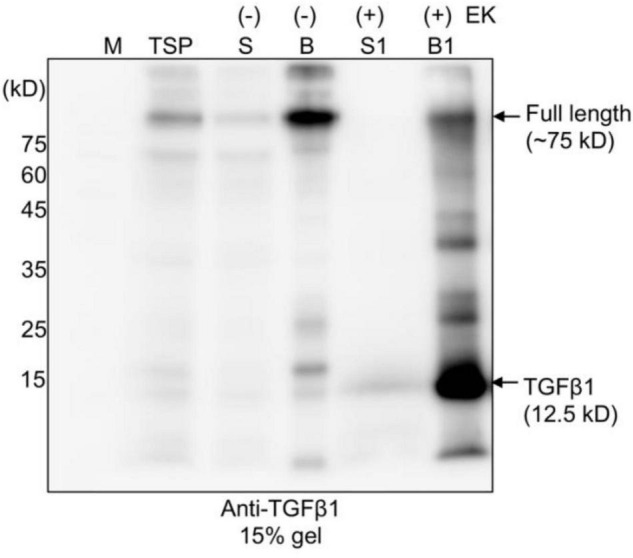
The full-length TGFβ1 recombinant protein is efficiently processed by enterokinase, but the mature form remains associated with the LAP domain. Full-length TGFβ1 recombinant proteins were purified using MCC beads and subsequently incubated with (+) or without (-) enterokinase at 25^°^C for 16 h. The incubation solutions (S and S1) and MCC beads (B and B1) were collected separately. The MCC beads were washed with 40 mM Tris-Cl (pH 7.4) to remove enterokinases. Proteins still bound to the MCC beads were released by boiling them in SDS/PAGE sample buffer. All protein samples were separated by SDS/PAGE and analyzed by Western blotting using an anti-TGFβ1 antibody. TSP, total soluble protein.

Next, as an alternative approach, we examined whether another protease, enterokinase (EK), could replace furin for the release of mature TGFβ1 from the full-length TGFβ1 recombinant protein. EK is a serine protease. The recombinant catalytic subunit is widely used for the cleavage of recombinant proteins that contain a recognition sequence, DDDDK (Gasparian et al., 2011). We replaced the furin cleavage site (RRHR) with an EK site (DDDDK) to yield construct *BMC:LAP[C33S]:EK:TGF*β*1* (Supplementary Figure 2). We first compared the expression levels of *BMC:LAP[C33S]:EK:TGF*β*1* and *BMC:LAP[C33S]TGF*β*1* in *N*. *benthamiana*. Total soluble protein extracts from the leaves of *N*. *benthamiana* transformed with *BMC:LAP[C33S]TGF*β*1* and *BMC:LAP[C33S]:EK:TGF*β*1* at 3, 5, and 7 DPI were separated by SDS/PAGE and analyzed by anti-TGFβ1 antibody. The replacement of the furin cleavage site with an EK site did not affect the expression level of the *TGF*β*1* recombinant gene (Supplementary Figures 2B,C). Next, we examined whether full-length TGFβ1 recombinant proteins could be cleaved by an enterokinase light chain. For this, we first purified BMC:LAP[C33S]:EK:TGFβ1 using MCC beads. The MCC beads bound with recombinant protein BMC:LAP[C33S]:EK:TGFβ1 were incubated with EK. Proteins in the digestion buffer and proteins bound to MCC beads were separately collected and analyzed by SDS/PAGE and Western blotting using an anti-TGFβ1 antibody. A protein band at 12.5 kD corresponding to monomeric mature TGFβ1 was detected only with EK (Figure 3). Moreover, BMC:LAP[C33S]:EK:TGFβ1 was cleaved by EK with over 90% efficiency, as calculated based on western blot band intensity, indicating that the recombinant proteins of TGFβ1 were correctly and efficiently processed by EK. However, the 12.5 kD band was detected in proteins from the MCC bead fraction but not in the EK buffer fraction, indicating that the mature form of TGFβ1 was not released from the LAP domain, consistent with earlier results showing that mature TGFβ1 is tightly associated with LAP after cleavage.

### Mature TGFβ1 Is Released From LAP *via* Activation With HCl Treatment

**FIGURE 4:**
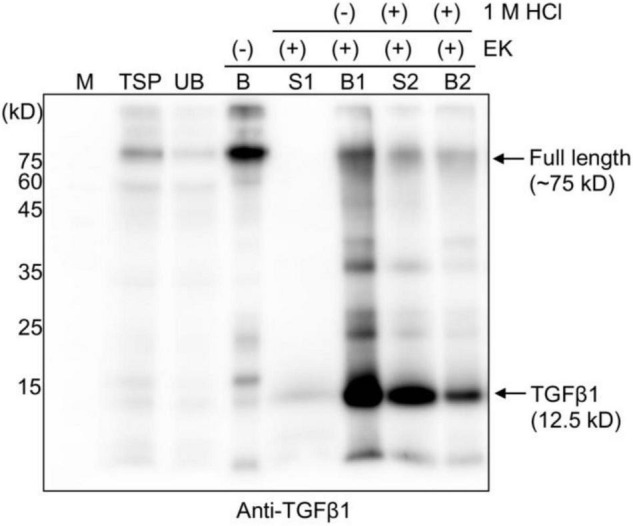
HCl treatment releases mature TGFβ1 from the LAP domain after enterokinase cleavage. Total soluble protein (TSP) extracts were mixed with MCC beads for 2 h at 4^°^C. The supernatant and MCC beads were separated into unbound (UB) and MCC bead-bound (B) fractions. The MCC bead fraction was washed four times with washing buffer (40 mM Tris-Cl, pH 7.4, 0.05% Triton X-100). Subsequently, MCC beads were suspended in EK buffer (50 mM Tris-Cl, pH 7.4, 150 mM NaCl, 2 mM CaCl_2_, and 0.5% Triton X-100) and incubated with EK at 25^°^C for 16 h. The supernatant (S1) and MCC beads (B1) were collected separately. The MCC beads (B1) carrying the complex of mature TGFβ1 and BMC-LAP were mixed with activation buffer (40 mM Tri-Cl, pH 7.4, and 150 mM NaCl), and 1 M HCl was added at 1/20th the volume (V/V) of activation buffer to give 50 mM HCl as the final concentration and incubated in a shaker at 25^°^C for 30 min. The supernatant (S2) and MCC beads (B2) were collected separately. Proteins bound to the MCC beads were recovered by boiling them in SDS/PAGE sample buffer. Proteins were separated by 15% SDS/PAGE and analyzed by Western blotting using an anti-TGFβ1 antibody. M, molecular weight standard; arrows, the position of full-length or cleaved TGFβ1.

It is well-known that mature TGFβ1 is tightly associated with the LAP domain after cleavage by Furin in mammalian cells to regulate the availability of active TGFβ1 (Khalil, 1999). We asked how mature TGFβ1 could be released from the LAP after cleavage. Another question was how mature TGFβ1 could be activated after release from the LAP domain. In fact, the release and activation of TGFβ1 from LAP has been a real challenge, which makes the production of active TGFβ1 as a recombinant protein very difficult. Concerning the activation of TGFβ1, TGFβs are secreted in a biologically latent form, either smaller L-TGFβ or large LL-TGFβ, and none of them can interact with TGFβ receptors (Miyazono et al., 1991; Bonewald, 1999). Latent TGFβs can be activated *in vitro* by physiochemical factors, such as a low pH of 2 or a high pH of 8, a high temperature of 100^°^C, urea, detergents, such as SDS, and chaotropic agents (Brown et al., 1990). In addition, a few proteases, such as plasmin, calpain, neuraminidase, cathepsins B and D, and thrombospondin-1, activate latent TGFβ1 (TSP-1) (Miyazono et al., 1991; Schultz-Cherry et al., 1995; Khalil, 1999). A previous study showed that latent TGFβ1 is converted to the active form upon treatment with a low pH of 3.7 at 37^°^C in a time-dependent manner, yielding a significant portion or the maximum with incubation for only 15 min or 120 min, respectively (Nocera and Chu, 1995). The released TGFβ1 remained relatively stable for 24 h at the pH and temperature mentioned above. We examined whether mature TGFβ1 could be released from LAP at a low pH. Full-length recombinant proteins of TGFβ1 bound onto MCC beads were treated with EK, and the MCC beads were washed four times with 40 mM Tris-Cl (pH 7.4) buffer to remove EK and other non-specific proteins released from MCC beads. Subsequently, the MCC beads were incubated in an activation buffer with a pH of approximately 1.5–2 for 30 min at 25^°^C. Proteins released into the incubation solution were collected separately. Proteins bound to MCC beads were also collected by boiling the MCC beads in the SDS/PAGE sample buffer. These proteins were separated by SDS/PAGE and analyzed by Western blotting using an anti-TGFβ1 antibody. A major portion of mature TGFβ1 was detected in the solution fraction (Figure 4, lane S2), indicating that low pH treatment leads to the release of mature TGFβ1 from the LAP domain. A previous study suggested that a low pH causes denaturation of LAP but not TGFβ (Lawrence, 1995), thereby disturbing the interaction between them.

**FIGURE 5:**
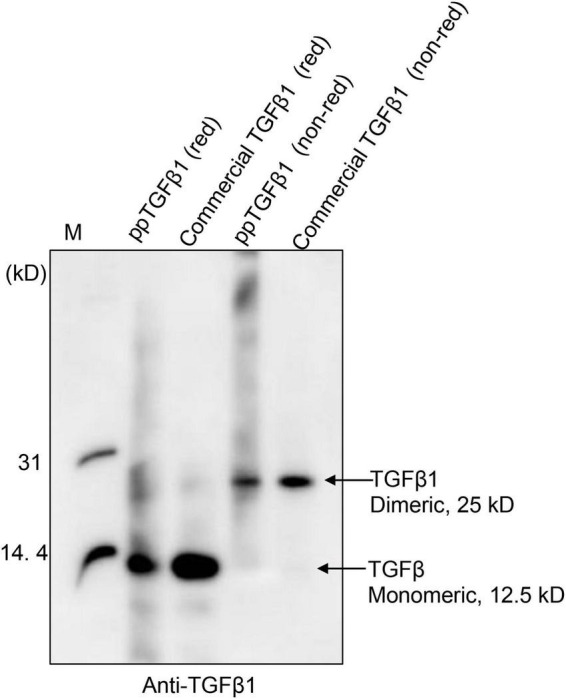
Mature TGFβ1 is released as dimers from the full-length TGFβ1 recombinant protein *via* EK-mediated proteolytic cleavage, followed by acid activation. The mature TGFβ1 released from the full-length TGFβ1 recombinant proteins by HCl treatment was separated by 10% Tricin/PAGE under reducing or non-reducing conditions and analyzed by Western blotting using an anti-TGFβ1 antibody. Commercial mature TGFβ1 (50 ng) produced in HEK293 cells was included as a positive control.

Next, we examined whether the mature TGFβ1 released by low pH treatment could yield the dimer form of mature TGFβ1. Proteins were separated by non-reducing tricin/PAGE without denaturation using DTT and analyzed by Western blotting using anti-TGFβ1 antibodies. As a control, commercial TGFβ1 was included in the analysis. Mature TGFβ1 was detected at 25 kD, corresponding to the dimer position (Figure 5 and Supplementary Figure 3), indicating that mature TGFβ1 exists as a dimer.

### Contaminating Proteins in Mature TGFβ1 Preparation Are Removed by a Second Microcrystalline Cellulose Bead Binding

**FIGURE 6:**
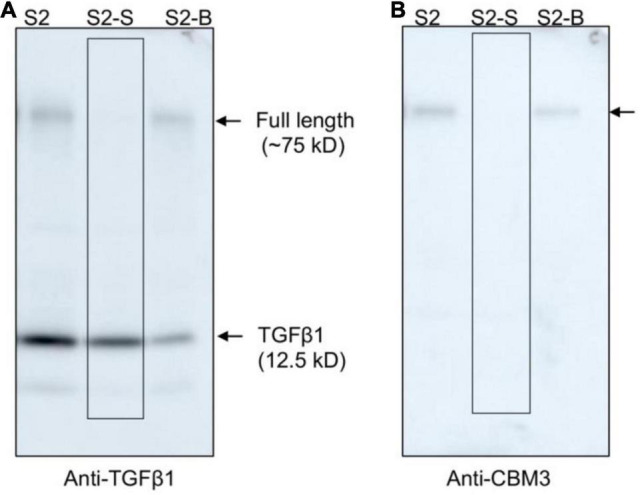
Full-length TGFβ1 recombinant proteins and other contaminating proteins can be removed from mature TGFβ1 by rebinding to MCC beads. The pH of the mature TGFβ1 preparation (S2) obtained after HCl treatment was adjusted by adding 40 mM Tris base (pH ∼11.0) until the pH reached 4.0. Subsequently, the mature TGFβ1 preparation was mixed with MCC beads and incubated on a twister at 4^°^C for 2 h. The mixture was centrifuged at 2,000 × g for 5 min, and the supernatant (S2-S) and MCC beads (S2-B) were recovered separately. Proteins bound to MCC beads were recovered by boiling them in SDS sample buffer. Protein samples were separated by SDS/PAGE and analyzed by Western blotting using anti-TGFβ1 **(A)** and anti-CBM3 **(B)** antibodies. Arrows, the position of full-length or mature TGFβ1.

The mature TGFβ1 preparation obtained by HCl treatment was contaminated with the uncleaved full-length TGFβ1 recombinant proteins together with a few degradation products. We wanted to further purify mature TGFβ1 to a higher degree of purity. CBM3 is known to bind tightly to MCC beads. However, the HCl treatment caused the release of the full-length recombinant proteins together with the degradation products. Since these contaminating proteins still contained CBM3, we asked whether they could again bind to MCC beads under mild pH conditions. For this, the pH of the mature TGFβ1 preparation obtained from MCC beads was adjusted to a pH of 4 by adding 40 mM Tris-base (pH ∼11), mixed with MCC beads, and incubated for 2 h. The mixture was separated into supernatant and MCC bead fractions. Proteins in the MCC beads were obtained by boiling them in SDS/PAGE sample buffer. These protein samples were separated by SDS/PAGE and analyzed by Western blotting using an anti-TGFβ1 antibody. In this rebinding process, the majority of contaminating proteins were detected in the MCC bead fraction (S2-B fraction), and TGFβ1 was largely detected in the supernatant fraction (S2-S fraction) (Figures 6A,B), indicating that the majority of contaminating proteins could be eliminated by rebinding to MCC beads. However, some of the mature TGFβ1 protein was lost during this purification step (S2-B fraction).

Endotoxin-free recombinant proteins are one of the most critical advantages of using plants as hosts for recombinant protein production. Lipopolysaccharides (LPS) have always been an important concern when recombinant proteins are produced in *E*. *coli*. To deliver the recombinant gene into plant cells, we used *Agrobacterium*-mediated transformation. Thus, we examined the level of endotoxins, if any, in purified mature TGFβ1. Purified mature TGFβ1 preparation contained <0.013 EU/μg (<0.0013 ng/μg) endotoxin (Supplementary Figure 4), which is much lower than the acceptable limit of endotoxins in recombinant proteins, <1 EU/μg (<1 ng/μg) (Magnusdottir et al., 2013; Nomura et al., 2017).

### Plant-Produced Mature TGFβ1 Activates Downstream Signaling and Induces Filopodia-Like Structures in the A549 Cell Line

**FIGURE 7:**
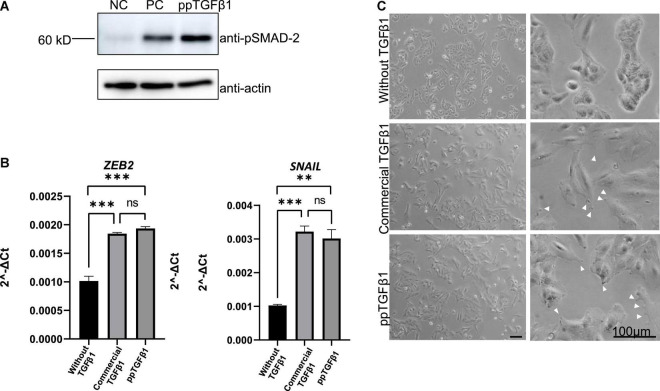
The mature TGFβ1 produced in plants activates the downstream signaling of TGFβ1 in the A549 cell line. **(A)** Phosphorylation of SMAD2. Total proteins were prepared from the A-546 cell line and treated with plant-produced TGFβ1 (ppTGFβ1) or commercial TGFβ1 (PC) at a concentration of 10 ng/ml concentration for 48 h. Proteins were separated by SDS/PAGE and analyzed with Western blotting using anti-pSMAD or anti-actin antibodies. NC, negative control (4 mM HCl). **(B)** qRT-PCR analysis of upregulated EMT pathway-specific genes. Total RNA was prepared from the A-546 cell line treated as in panel **(A)**. qRT-PCR was performed using primers for *ZEB2-* or *SNAIL*-specific primers. *GAPDH* was used as an internal control. qRT-PCR was performed with three independent biological samples, and statistical analysis was performed using an unpaired *t*-test (GraphPad Prism 9). The values are the means with standard deviations (*n* = 3). **(C)** Microscopic image of the A-546 cells. The cells were treated as in panel **(A)**. Images of cells were taken 48 h after treatment with plant-produced TGFβ1 or commercial TGFβ1. Arrows, filopodia. Scale bar = 100 μm. **P < 0.05 and ***P < 0.001.

We next examined whether plant-produced mature TGFβ1 was biologically active. TGFβ1 facilitates the oligomerization of Ser/Thr receptor kinases and phosphorylates cytoplasmic signaling molecules SMAD2 and SMAD3 (Schmierer and Hill, 2007). The very first step in TGFβ1 signaling is the binding of the ligand to dimers of receptor TβRII, which leads to oligomerization with TβRI to yield a heterotetrameric complex (Wrana et al., 1992). This interaction leads to a cascade of events and phosphorylates SMAD2 and SMAD3 proteins (Albers et al., 2018). We compared the activity of plant-produced TGFβ1 (ppTGFβ1) with commercial recombinant TGFβ1 produced in HEK293 cells. Human lung cancer cell A549 was cultured with or without ppTGFβ1 or commercial TGFβ1 (10 ng/ml) for 48 h. We measured the phosphorylated SMAD2 (p-SMAD2) levels. Upon treatment with commercial TGFβ1, the p-SMAD2 level dramatically increased in the A549 cell line. The ppTGFβ1 treatment also increased the p-SMAD2 level to a level higher than that with commercial TGFβ1 (Figure 7A), indicating that ppTGFβ1 is as active as commercial TGFβ1. To support this finding, we examined the activation of downstream genes. It is well-known that the epithelial–mesenchymal transition (EMT) pathway is upregulated upon TGFβ1 treatment (Liu et al., 2013). Two genes, *SNAIL1* and *ZEB2*, are known as TGFβ1 downstream signaling genes. qRT-PCR revealed that treatment with both commercial TGFβ1 and ppTGFβ1 rapidly enhanced *SNAIL1* transcript levels in A549 cells to the same degree (Figure 7B). Consistent with this, the transcript level of zinc-finger E-box-binding 2 (*ZEB2*), a transcription factor activated by SNAIL1, also increased upon treatment with both commercial TGFβ1 and ppTGFβ1 to the same degree. Finally, we examined the morphological changes in A549 cells. Actin reorganization is a prominent morphological alteration induced by TGFβ1, leading to lamellipodia/filopodia formation. In the absence of TGFβ1, the A549 cells showed regular shapes. However, treatment with both commercial TGFβ1 and ppTGFβ1 induced EMT-like elongated structures and filopodia formation (Figure 7C). These results confirm that ppTGFβ1 is as active as commercial TGFβ1.

## Discussion

In this study, we developed an efficient purification and one-pot activation method to produce active human TGFβ1 in *N*. *benthamiana*. Often, the production of recombinant proteins includes many rounds of purifications and downstream processing that can lead to not only an increased production cost but also a loss of yield. Our approach did not include tedious and complicated steps of refolding after purification, as in *E*. *coli*. To our knowledge, this is the first time that active dimeric TGFβ1 has been produced and purified in plants without the refolding process.

Recombinant human TGFβ1 was produced in heterologous systems, such as *E*. *coli* and HEK293 cell lines. In *E*. *coli*, the mature form of TGFβ1 was expressed. However, it was expressed as an inclusion body in *E*. *coli*, which requires multiple rounds of denaturation and a refolding process to yield active mature TGFβ1. In contrast, active TGFβ1 was successfully produced in CHO cells without the refolding process (Zou and Sun, 2004). In their study, LAP[C33S]TGFβ1 was expressed as a secretory protein using the leader sequence of rat serum albumin, one of the most abundantly secreted proteins in cells, to enhance the targeting of recombinant protein to the ER. The secreted protein was purified by Ni^2+^-NTA affinity purification followed by low pH (pH 3.0) activation, thereby releasing TGFβ1 from its LAP domain. Finally, TGFβ1 was further purified by size-exclusion chromatography at a yield of approximately 20 mg/l.

In a previous study, an attempt was made to produce TGFβ1 in plants. When LAPTGFβ1, the pro-form of TGFβ1, was co-expressed with *furin*, active TGFβ1 was produced (Wilbers et al., 2016). However, the expression level was too low for the purification of mature TGFβ1. The activity was tested using acid-activated total soluble protein and ELISA. In this study, we also used the pro-form of TGFβ1, LAP[C33S]TGFβ1, to produce TGFβ1 in *N*. *benthamiana*. In the production of recombinant proteins in heterologous systems, one of most important aspects is the expression level. We used the M domain to increase the expression level of TGFβ1. The M domain has been shown to increase the protein expression level when the fusion protein is targeted at the ER (Kang et al., 2018). In addition, the stability of recombinant proteins is crucial to achieving a high yield. In plants, the ER is the best place to produce recombinant proteins because it provides a suitable environment for correct folding and post-translational modification and is the place with the least risk of proteolytic degradation (Schouten et al., 1996). Often, an ER retention motif is added to the C-terminus for ER accumulation. However, it has been shown that the addition of extra amino acid residues to the C-terminus of TGFβ1 hampers biological activity (Wakefield et al., 1991). Thus, we did not add the ER retention motif to the C-terminus of the TGFβ1 recombinant protein. This could be a limiting factor in increasing the expression level. In plants, another member of the TGFβ family, TGFβ3, was successfully produced in *N*. *tabacum* (Gisby et al., 2011). To produce TGFβ3 at a high level in plants, the authors integrated the gene encoding the mature form of TGFβ3 into the chloroplast genome. Indeed, this approach is one of the most powerful ways to increase expression levels in plants. However, the mature form of TGFβ3 was produced as an inclusion body, which necessitates denaturation and refolding, as in the case of TGFβ1 in *E*. *coli*.

Another important step in the production of recombinant proteins is protein purification from the total protein extracts of plant leaf tissues. Indeed, it has been estimated that the purification steps account for 70–80% of the production costs (Raven et al., 2014). Various fusion tags have been introduced to improve protein purification, recovery, and solubility (Loughran and Walls, 2017). Recently, cellulose-binding module 3 (CBM3) has been used as an affinity purification tag due to its high selectivity and tight binding to cellulose, which is an inexpensive material (Wan et al., 2011; You and Zhang, 2013; Islam et al., 2018b). The binding affinity between CBM3 and MCC beads is extremely high and can only be disrupted by strong denaturants (Sarikaya et al., 2003; Andrade et al., 2010). We included CBM3 as an affinity tag for protein purification of the TGFβ1 recombinant protein. Indeed, BMC:LAP[C33S]TGFβ1 tightly bound to MCC beads. Moreover, the recombinant protein BMC:LAP[C33S]:EK:TGFβ1 bound to the MCC beads was efficiently cleaved by EK. In this process, furin, an endogenous processing enzyme, was less efficient in processing BMC:LAP[C33S]TGFβ1 bound to MCC beads. However, the mature form of TGFβ1 was not released from MCC beads even after EK treatment. One possibility is that mature TGFβ1 may be tightly associated with LAP[C33S] as reported previously (Khalil, 1999). This behavior of mature TGFβ1 is advantageous in purifying mature TGFβ1 after EK cleavage. MCC beads can be washed to remove EK after the cleavage reaction.

The release of active mature TGFβ1 from LAP is tightly controlled in cells and induced by signaling *in vivo* (Shi et al., 2011). Thus, in the production of TGFβ1 as a recombinant protein, the release and activation of TGFβ1 from the LAP domain is challenging. Various treatments, including high salt (1 M NaCl), urea (1–4 M), detergent (0.5% SDS), DTT, and low pH buffers (glycine pH 3.0), did not induce the release of mature TGFβ1 from the LAP domain (del Amo-Maestro et al., 2019). We found that treatment with a high concentration of HCl successfully released mature TGFβ1 from the LAP domain in dimeric form after EK cleavage. The release of TGFβ1 was specific to a high concentration of HCl but not to other acidic conditions, such as 0.2 M glycine (pH 2.0). This raises the possibility that an acidic pH, together with Cl^–^, but not acidic conditions *per se*, plays a role in the release of mature TGFβ1 from the LAP domain. However, this needs to be further investigated.

Recently, recombinant TGFβ1 was produced in mammalian cells such as CHO cells. However, in general, recombinant protein production in animal cells requires a highly sophisticated facility that should be run under aseptic conditions. Also, the running cost is high, which leads to a high price of the final product. In contrast, plants as a bio-factory give great freedom to such situations. The facility and running cost are thought to be much lower than those for animal cells. An additional advantage is that recombinant proteins produced in plants are free from animal pathogens. In this study, we explore this possibility using TGFβ1, one of the challenging targets in recombinant protein production. The pure TGFβ1 that could be obtained in our current study was up to 1 mg/kg of fresh weight. In the case of the CHO cells, the amount of protein produced was up to 30 mg/L. The yield *per se* appears to be lower in *N. benthamiana* than in CHO cells. However, considering the high running cost and expansive facility, for the production of recombinant TGFβ1, we believe that the plant system may still be competitive to the CHO system. In addition, there are still rooms to enhance the expression level of recombinant TGFβ1 using strong expression vectors such as virus-based expression vectors. As the current study employed simple and inexpensive steps for purification, it would definitely reduce the overall cost of the final product once higher expression is achieved. We demonstrate that dimeric active TGFβ1 can be produced in plants and purified using cheap MCC beads to a high degree of purity without using time-consuming and costly processes, such as denaturation and refolding *in vitro*. The purity of the final TGFβ1 product can be further improved by additional steps involving size exclusion, ion exchange, or hydrophobic interaction chromatography. In conclusion, this plant-based system is a low-cost production system for dimeric active TGFβ1 that can be used for pharmaceutical purposes.

## Materials and Methods

### Recombinant Gene Constructs

Codon-optimized *LAPTGF*β*1* (without signal peptide, GenBank NM_000660) was chemically synthesized (Bioneer Corp., Daejeon, South Korea). *TGF*β*1* was amplified with PCR from *LAPTGF*β*1* using *Bam*HI_6xHis_TGFβ1 forward primer and *Xho*I_LAP_TG_R2 reverse primer (Supplementary Table 1). The PCR product was digested by *Bam*HI and *Xho*I restriction endonucleases and ligated to *pTEX1-BiP:HA:mCor1:LysM:His:HDEL* (Song et al., 2021) to generate *pTEX1-BiP-6xHis-TGF*β*1*. The *M* domain and *CBM3* were amplified with PCR from *1300-BMC-SazCA* (Kumari et al., 2020). *Bam*HI and *Spe*I restriction sites were introduced in the *M* domain by overhang PCR using primers MF and MR (Supplementary Table 1). To introduce an *Spe*I site to the 5’ end, and *Xma*I and *Xho*I sites to the 3’ end of *CBM3*, PCR was carried out using primers CBM_F and CBM_R (Supplementary Table 1).

The PCR product *Bam*HI-M-*Spe*I was digested with *Bam*HI and *Spe*I restriction endonucleases and ligated to *pTEX1*-*BiP:HA:mCor1:LysM:His:HDEL* (Song et al., 2021) to generate *pTEX1-BM*. The PCR product, *Spe*I-CBM3-*Xma*I-*Xho*I, was digested with *Spe*I and *Xho*I restriction endonucleases and ligated to *pTEX1-BM* digested by *Spe*I and *Xho*I to produce another intermediate vector, *pTEX1-BMC*. Chemically synthesized *LAPTGF*β*1* was digested by *Xma*I and *Xho*I restriction endonucleases and ligated to *pTEX1-BMC* digested with the same restriction endonucleases to yield *pTEX1-BMC:LAPTGF*β. [C33S] mutation in the LAP domain was introduced by overhang PCR using forward primer *Xma*I_GG_LAPC33S and reverse primer *Xho*I_LAP TG_R2. The PCR product was digested with *Xma*I and *Xho*I restriction endonucleases and ligated to *pTEX1-BMC:LAPTGF*β*1*, digested with the same restriction endonucleases to yield *pTEX1-BMC:LAP[C33S]TGF*β*1.* To replace the furin cleavage site with an enterokinase cleavage site (DDDDK), overlap PCR was performed using four primers, *Xma*I_LAP-TG_F1, Fu: EK_OL_R1, Fu: EK _OL_F2, *Xho*I_LAP_TG_R2 (Supplementary Table 1), and the PCR product was digested with the *Xma*I and *Xho*I restriction endonucleases and ligated to *pTEX1-BMC:LAP[C33S]TGF*β*1* digested with the same restriction endonucleases to yield *pTEX1-BMC:LAP[C33S]:EK:TGF*β.

### Plant Growth Conditions

Wild-type *Nicotiana benthamiana* plants were grown in a controlled greenhouse. The temperature and relative humidity were controlled at 24^°^C and 40–65%, respectively. The photoperiod was adapted to a long-day photoperiod (14 h light and 10 h dark; light intensity, 130–150 μE/m^2^) for 5–7 weeks. Five- to seven-week-old plants were used for agro-infiltration.

### Transient Expression of Recombinant *TGFβ1* in *Nicotiana benthamiana*

All expression vectors were transformed into *Agrobacterium tumefaciens* strain EHA105 by electroporation and plated on an LB-agar plate containing 50 mg/ml kanamycin and 50 mg/ml rifampicin. A single colony was used to inoculate in 5 ml LB medium and cultured overnight. A 5 ml culture was added to 50 ml LB medium containing suitable antibiotics. Cells were pelleted by centrifugation at 3,500 × g for 10 min and resuspended in infiltration buffer (10 mM MES, 10 mM MgSO_4_, 200 μM acetosyringone, pH 5.6) at 0.8 OD_600_. The cell suspension was maintained at room temperature for 2–4 h before infiltration. Leaves of 5–7-week-old plants were infiltrated on the abaxial side using a 1 ml syringe without a needle or by vacuum infiltration. Infiltrated plants were returned to the greenhouse for further growth of 3–7 days.

Leaf samples were harvested, ground in liquid nitrogen using a mortar and pestle to give fine powder and mixed with 5 volumes (w/v) of protein extraction buffer (50 mM Tris-Cl, pH 7.4, 300 mM NaCl, 1 mM DTT, 0.1% [v/v] Triton X-100, and 1X protease inhibitor cocktail). The total soluble protein was recovered after centrifugation at 13,000 × g for 15 min. Protein concentrations were measured using the Bradford protein method (Bio-Rad, Hercules, CA, United States).

### SDS-PAGE and Western Blot Analysis

Protein samples were separated using 10–15% SDS/PAGE. Gels were stained with 0.25% CBB R-250 (AMRESCO, cat. no: 6104-59-2) in a staining solution containing 45% methanol and 10% glacial acetic acid or analyzed by Western blotting with suitable antibodies.

For Western blot analysis, membranes were blocked with 5% fat-free skim milk in TBST buffer (20 mM Tris-Cl, pH 7.5, 150 mM NaCl, 0.1% Tween-20) for 2 h and incubated with rabbit anti-TGFβ1 (Abcam, ab179695), rabbit anti-CBM3 (Bio app., South Korea), anti-phospho-SMAD2 (AB3849-I Merck, Rahway, New Jersey, United States), or anti-actin-clone C4 (Merck, Rahway, New Jersey, United States) antibodies as the primary antibody at a dilution of 1:1,000–1:5,000 in TBST with 5% non-fat dry milk overnight followed by washing and incubation with respective secondary antibodies at a dilution of 1:5,000–1:10,000 in TBST at room temperature for 2 h. Immunoblot images were captured using an Amersham Imager 680 (GE Healthcare, Chicago, IL, United States).

### Protein Purification Using Microcrystalline Cellulose Beads and Microcrystalline Cellulose Bead-Bound Cleavage of Full-Length TGFβ1 Recombinant Proteins by Enterokinase

To purify BMC:LAP[C33S]:EK:TGFβ1, total soluble protein (TSP) extracts were mixed with an MCC bead (Sigma Aldrich, Burlington, MA, United States) slurry in batch. First, the MCC bead slurry was prepared by mixing MCC bead powder with autoclaved distilled water at a 1:1 (w/v) ratio. The MCC beads were washed five times to remove very fine particles. Finally, an equal volume of sterile water was added to the MCC beads, and the MCC bead slurry was maintained at 4^°^C for future use. TSP extracts (150 ml) from 30 g FW leaf tissues were mixed with 8 ml MCC slurry and incubated at 4^°^C for 2 h with gentle shaking. After the binding of proteins to MCC beads, samples were centrifuged at 2,000 × g for 2 min, and the supernatant and MCC beads were separately collected for unbound proteins and MCC bead-bound proteins, respectively. The recovered beads were washed five times with 10 bead volumes of 40 mM Tris-HCl buffer (pH 7.4, 0.05% Triton X-100) to remove loosely bound proteins. MCC bead-bound proteins were released by boiling them in SDS/PAGE sample buffer, and the purity of MCC bead-purified TGFβ1 recombinant proteins was examined by SDS/PAGE and Western blot analysis.

To remove the N-terminal region from the mature TGFβ1, MCC beads bound with BMC:LAP[C33S]:EK:TGFβ1 recombinant proteins were first washed with enterokinase (EK) buffer (50 mM Tris-HCl, pH 7.4, 150 mM NaCl, 2 mM CaCl_2_, and 0.5% Triton X-100) and then divided into two 50 ml conical tubes at equal volumes. EK (30 units in 4 ml EK cleavage buffer) was added to the MCC beads bound with TGFβ1 recombinant proteins, and the mixture was incubated at 25^°^C in a shaking incubator for 16 h.

### Release and Activation of Mature TGFβ1 From the LAP Domain and Purification of Mature TGFβ1

After EK-mediated proteolytic cleavage of BMC:LAP[C33S]:EK:TGFβ1, MCC beads were centrifuged at 3,000 × g for 5 min, and the pelleted MCC beads were collected and washed twice with 5 ml of 40 mM Tris-HCl (pH 7.4). For the release and activation of mature TGFβ1, TGFβ1/BMC:LAP[C33S]-bound MCC beads were suspended in 5 ml activation buffer (40 mM Tri-HCl pH 7.4, and 150 mM NaCl). After gentle mixing, 1 M HCl at 1/20th the volume of activation buffer was added to TGFβ1/BMC:LAP[C33S]-bound MCC beads to give a final HCl concentration of 50 mM. The mixture was incubated at 25^°^C for 30 min with gentle mixing. The mature form of TGFβ1 was recovered in the supernatant after centrifugation at 3,000 × g at 4^°^C for 5 min. To recover any remaining mature TGFβ1 from MCC beads, 2 ml activation buffer and 100 μl of 1 M HCl were added to the MCC beads, gently mixed for 1 min, and the supernatant was collected after centrifugation. The pH of the pooled supernatant was increased to 4.0 by adding 40 mM Tris base (pH ∼11.0). The concentration of NaCl was readjusted to get final concentration 150 mM.

To further purify the mature TGFβ1, the supernatant was mixed again with 1 ml of the MCC bead slurry and incubated for 2 h. The mature TGFβ1 was recovered from the supernatant after centrifugation at 3,000 × g for 5 min. Purified TGFβ1 was extensively dialyzed against 4 mM HCl and subjected to centrifugation at 14,000 × g for 20 min, and the supernatant containing mature TGFβ1 was recovered. The dialyzed protein was concentrated using a Millipore 10 K centrifugal filter (Millipore, Burlington, MA, United States), and BSA was added to a 0.1% final concentration. The protein sample was filter-sterilized and stored at –80^°^C.

### Endotoxin Level Determination

The endotoxin level in purified mature TGFβ1 (ppTGFβ1) was determined using a Toxisensor Chromogenic LAL Endotoxin Assay Kit (GenScript, Cat no. L00350C, NJ, United States) according to the manufacturer’s protocol.

### Biological Activity of Plant-Produced Mature TGFβ1

Animal cell line A549 was purchased from the Korean Cell Line Bank (KCLB). Cells were seeded in 6-well plates (2.0 × 10^5^) and cultured in RPMI 1640 medium (Welgene, Gyeongsan-si, South Korea) supplemented with 10% fetal bovine serum (FBS) (Merck, Darmstadt, Germany), 100 IU/mL penicillin, and 100 μg/mL streptomycin (Welgene, Gyeongsan-si, South Korea). After 24 h of incubation, the cells were treated with 10 ng/ml commercial mature TGFβ1 (Abcam, Cambridge, United Kingdom), 10 ng/ml ppTGFβ1, or 4 mM HCl as a negative control and incubated at 37°C in 5% CO_2_ for 48 h.

### Real-Time Quantitative PCR

The cells were detached by scrapers and collected. Total RNA was prepared using the RNeasy Mini Kit (Qiagen, Hilden, Germany) and purified using the Qiagen RNeasy Mini Kit. RNA concentration and purity were determined using a NanoDrop One spectrophotometer (Thermo Fisher Scientific, Waltham, MA, United States). cDNA was prepared from 2 μg total RNA using a high-capacity cDNA Reverse Transcription Kit (Applied biosystems, Waltham, MA, United States) with oligo-dT as the primer, according to the manufacturer’s instructions. Quantitative RT-PCR was performed using SYBR Green Supermix and a one-step cycler (Applied biosystems, Waltham, MA, United States). Gene expression was normalized to the housekeeping gene *GAPDH*. The following primer pairs were used for qRT-PCR: *ZEB2* (sense, 5′- GGC GCA AAC AAG CCA ATC CCA -3′; antisense, 5′- TTC ACT GGA CCA TCT ACA GAG GCT T -3′); *SNAIL* (sense, 5′- ACC CCA CAT CCT TCT CAC TG -3′; antisense, 5′- TAC AAA AAC CCA CGC AGA CA -3′). The 20 μl PCR mixture contained 200 ng template, 0.5 μM each of the forward and reverse primers, and 1 × SYBR master mix. The PCR conditions were as follows: initial denaturation at 95°C for 10 min, followed by 40 cycles of 95°C for 15 s and 60°C for 1 min. To confirm specific amplification, a melting curve was generated by heating at 95°C for 15 s, then at 60°C for 1 min, and increasing the temperature by 0.3°C every 15 s up to 95°C. Statistical analysis was performed using an unpaired *t*-test (GraphPad Prism 9).

## Data Availability Statement

The original contributions presented in the study are included in the article/Supplementary Material, further inquiries can be directed to the corresponding author.

## Author Contributions

IH and AS contributed to conceptualizing this study and wrote the manuscript. AS made the construct designs and performed the required experiments. JL and KS contributed to determining the biological activity of ppTGFβ1. HK contributed to designing a few of the experiments. All authors contributed to the article and approved the submitted version.

## Conflict of Interest

The authors declare that the research was conducted in the absence of any commercial or financial relationships that could be construed as a potential conflict of interest.

## Publisher’s Note

All claims expressed in this article are solely those of the authors and do not necessarily represent those of their affiliated organizations, or those of the publisher, the editors and the reviewers. Any product that may be evaluated in this article, or claim that may be made by its manufacturer, is not guaranteed or endorsed by the publisher.

## Supplementary Material

Supplementary Figure 1 Furin does not cleave the furin cleavage site located between mature TGFβ1 and LAP in the full-length TGFβ1 recombinant protein *in vivo*. **(A)** Schematic presentation of the constructs. The native and modified furin cleavage sites (RHRR and RERRRKKR in *BMC:LAP[C33S]TGF*β*1* and *BMC:LAP[C33S]:F2:TGF*β*1*, respectively) are indicated in the TGFβ1 recombinant constructs. For the Furin construct, the furin gene encoding amino acid positions from 26 to 595 was fused to the leader sequence of *Arabidopsis* BiP. His tag and ER retention signal HDEL were also added to the C-terminus of furin. **(B)** Failure of the full-length TGFβ1 recombinant protein by furin. *BiP:furin_26–595_:6xHis:HDEL* was co-expressed with two TGFβ1 recombinant constructs in *N*. *benthamiana*. Total soluble protein samples prepared at 3, 5, and 7 DPI were separated on 15% SDS/PAGE and analyzed by Western blotting using anti-TGFβ1 or anti-His antibodies. Arrows, full-length recombinant protein or cleaved TGFβ1.

Supplementary Figure 2 Replacement of the furin cleavage site for efficient proteolytic cleavage. **(A)** Schematic presentation of the constructs containing either the furin cleavage sequence (RHRR) or the enterokinase cleavage sequence (DDDDK). **(B,C)** The expression level of *BMC:LAP[C33S]TGF*β*1* and *BMC:LAP[C33S]:EK:TGF*β*1*. Total protein extracts from leaf tissues of *N*. *benthamiana* harvested at 3, 5, and 7 DPI were analyzed by SDS/PAGE and Western blotting using anti-TGFβ1 antibody. M, molecular weight standard; NT, non-transformed wild-type; Arrow, full-length recombinant protein.

Supplementary Figure 3 Treatment with HCl, but not with other acids, releases dimeric TGFβ1 after proteolytic cleavage. After EK-mediated cleavage, MCC beads bound with the TGFβ1/BMC:LAP complex were treated with various acids for 30 min at 25^°^C. The supernatants from the MCC beads were separated on 10% Tricin-PAGE and analyzed by Western blotting using anti-TGFβ1 antibodies. Only HCl treatment released the dimers of TGFβ1. In contrast, the citrate buffer largely produced monomers of TGFβ1. M, molecular weight standard; HCl, 1 M HCl; CB, 30 mM citrate buffer; Gly, 200 mM glycine pH 2.

Supplementary Figure 4 Plant-produced and purified mature TGFβ1 contains an extremely low level of endotoxin. The amount of endotoxin was measured by a chromogenic LAL-assay. **(A)** The standard curve was generated using a commercial *E*. *coli* endotoxin standard. **(B)** Varying amounts (1–4 μg) of purified ppTGFβ1 were used to determine the endotoxin level. The values are means with standard deviations (*n* = 3).
