# Supplementary figures and images for: Production of Recombinant Active Human TGFβ1 in Nicotiana benthamiana
Source: Front Plant Sci. 2022 May 31;13:922694. doi: 10.3389/fpls.2022.922694 (PMC9197560; doi:10.3389/fpls.2022.922694)

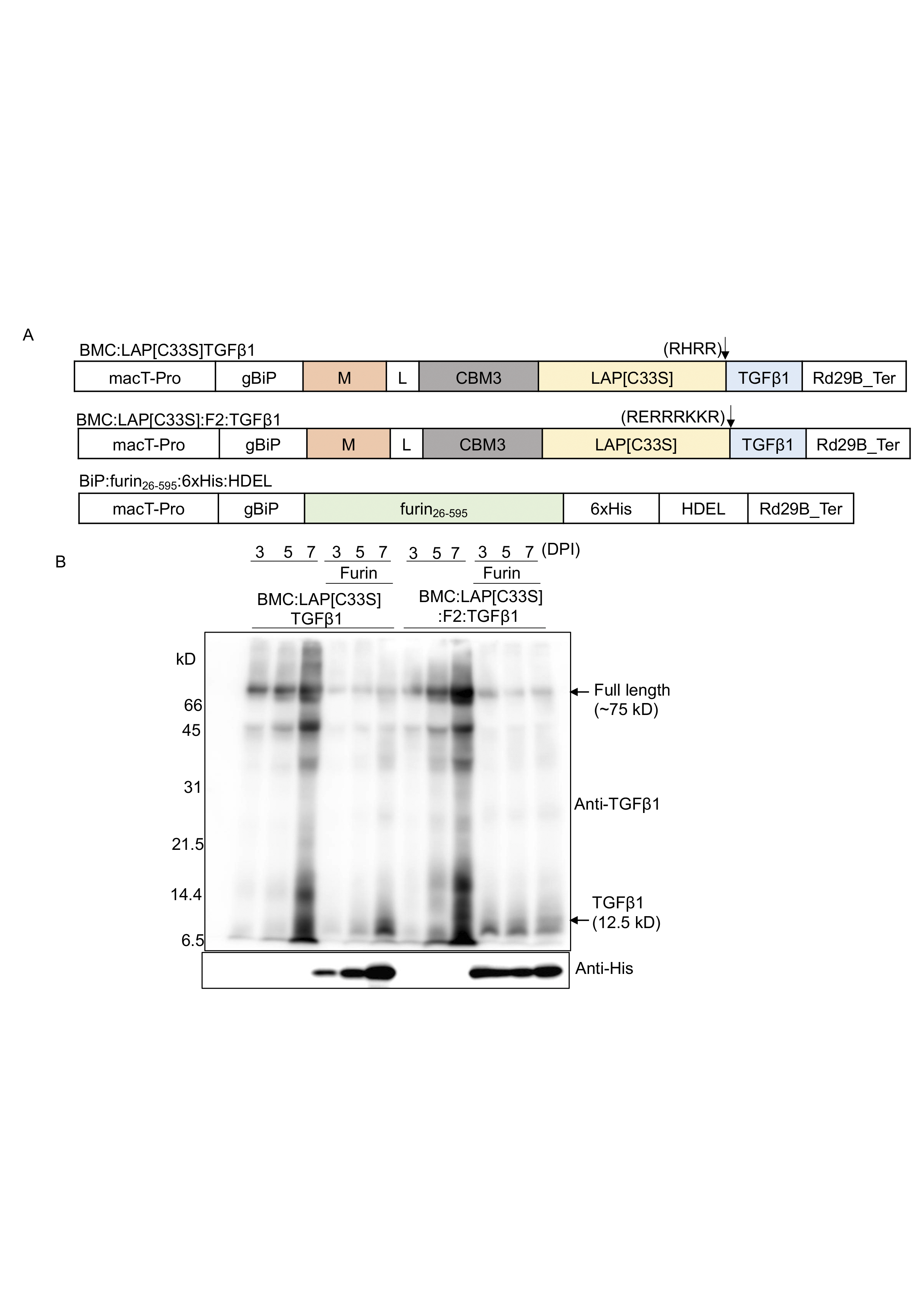

Supplement: Supplementary Figure 1 — Furin does not cleave the furin cleavage site located between mature TGFβ1 and LAP in the full-length TGFβ1 recombinant protein in vivo. (A) Schematic presentation of the constructs. The native and modified furin cleavage sites (RHRR and RERRRKKR in BMC:LAP[C33S]TGFβ1 and BMC:LAP[C33S]:F2:TGFβ1, respectively) are indicated in the TGFβ1 recombinant constructs. For the Furin construct, the furin gene encoding amino acid positions from 26 to 595 was fused to the leader sequence of Arabidopsis BiP. His tag and ER retention signal HDEL were also added to the C-terminus of furin. (B) Failure of the full-length TGFβ1 recombinant protein by furin. BiP:furin26–595:6xHis:HDEL was co-expressed with two TGFβ1 recombinant constructs in N. benthamiana. Total soluble protein samples prepared at 3, 5, and 7 DPI were separated on 15% SDS/PAGE and analyzed by Western blotting using anti-TGFβ1 or anti-His antibodies. Arrows, full-length recombinant protein or cleaved TGFβ1. [file Image_1.tif]

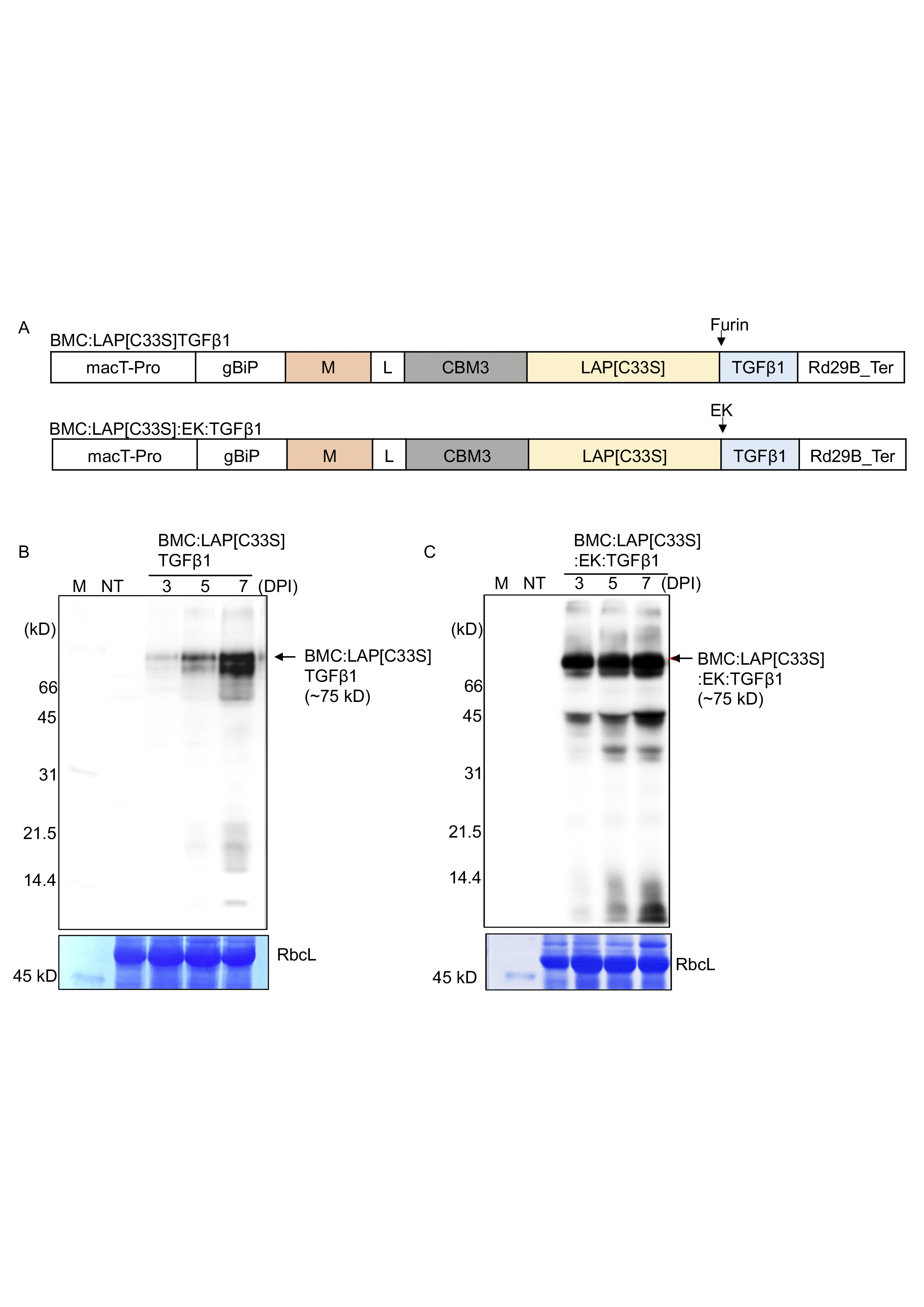

Supplement: Supplementary Figure 2 — Replacement of the furin cleavage site for efficient proteolytic cleavage. (A) Schematic presentation of the constructs containing either the furin cleavage sequence (RHRR) or the enterokinase cleavage sequence (DDDDK). (B,C) The expression level of BMC:LAP[C33S]TGFβ1 and BMC:LAP[C33S]:EK:TGFβ1. Total protein extracts from leaf tissues of N. benthamiana harvested at 3, 5, and 7 DPI were analyzed by SDS/PAGE and Western blotting using anti-TGFβ1 antibody. M, molecular weight standard; NT, non-transformed wild-type; Arrow, full-length recombinant protein. [file Image_2.tif]

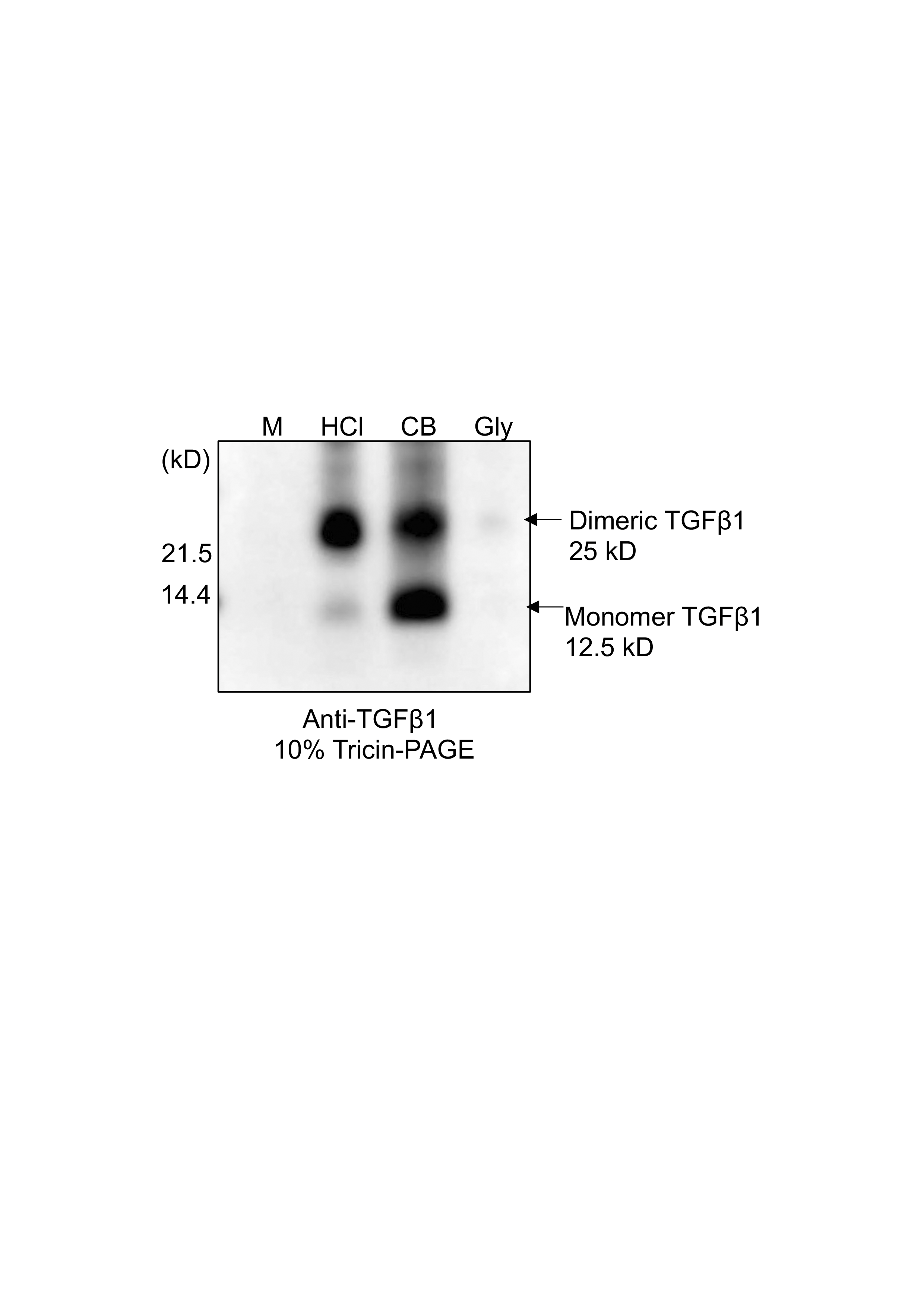

Supplement: Supplementary Figure 3 — Treatment with HCl, but not with other acids, releases dimeric TGFβ1 after proteolytic cleavage. After EK-mediated cleavage, MCC beads bound with the TGFβ1/BMC:LAP complex were treated with various acids for 30 min at 25°C. The supernatants from the MCC beads were separated on 10% Tricin-PAGE and analyzed by Western blotting using anti-TGFβ1 antibodies. Only HCl treatment released the dimers of TGFβ1. In contrast, the citrate buffer largely produced monomers of TGFβ1. M, molecular weight standard; HCl, 1 M HCl; CB, 30 mM citrate buffer; Gly, 200 mM glycine pH 2. [file Image_3.tif]

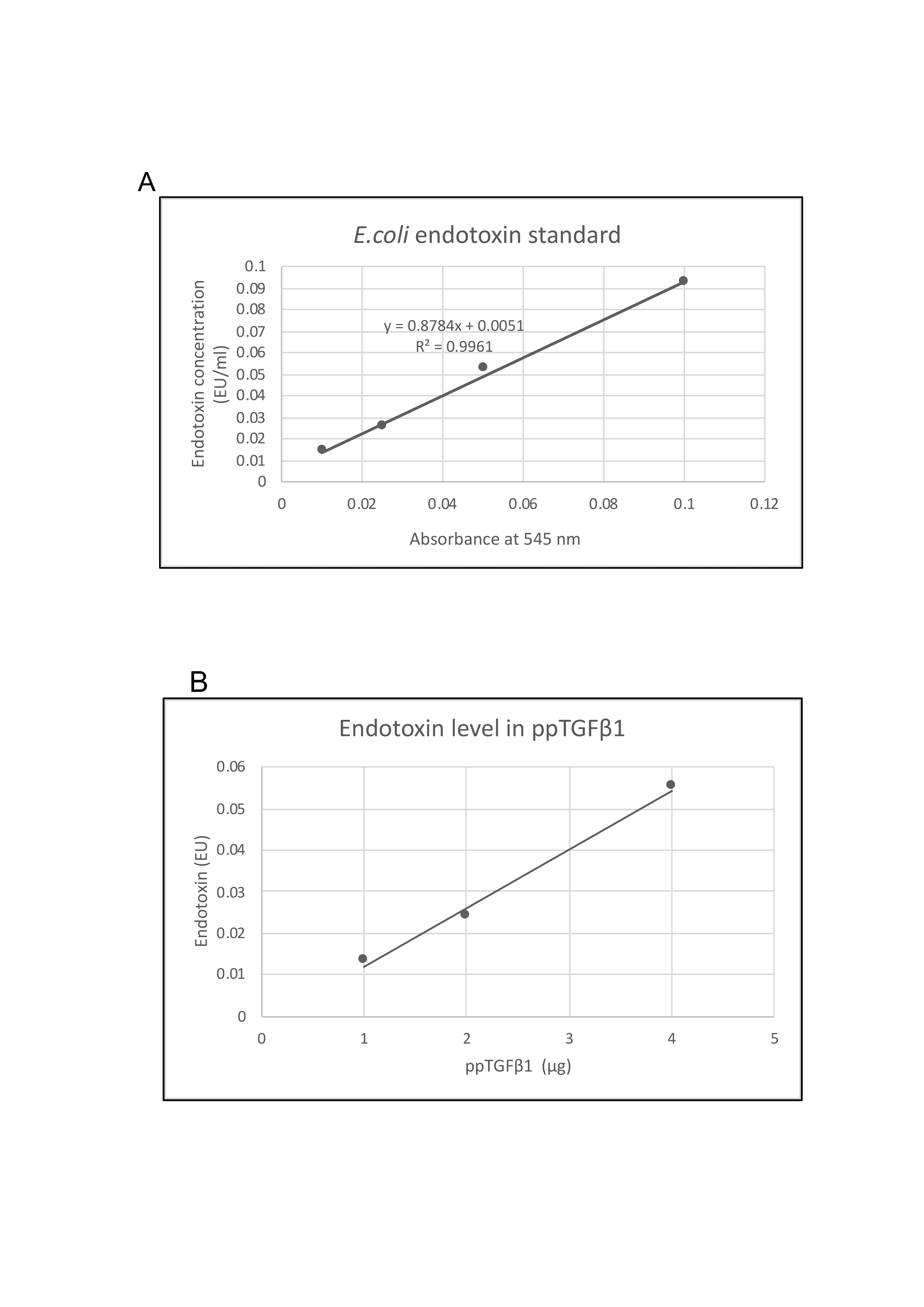

Supplement: Supplementary Figure 4 — Plant-produced and purified mature TGFβ1 contains an extremely low level of endotoxin. The amount of endotoxin was measured by a chromogenic LAL-assay. (A) The standard curve was generated using a commercial E. coli endotoxin standard. (B) Varying amounts (1–4 μg) of purified ppTGFβ1 were used to determine the endotoxin level. The values are means with standard deviations (n = 3). [file Image_4.tif]
