# Supplementary material for: Production of Recombinant Active Human TGFβ1 in Nicotiana benthamiana
Source: Front Plant Sci. 2022 May 31;13:922694. doi: 10.3389/fpls.2022.922694 (PMC9197560; doi:10.3389/fpls.2022.922694)
Supplement: Supplementary file 5 [file Table_1.pdf]

Supplementary Table 1. Nucleotide sequences of primers used in this study.

Primers; 5’ to 3’
BamHI_6xHis_TGFβ1: CGCGGATCCAACACCATCACCACCATCACGCTCTTGACACTAATTATTG
MF: CGCGGATCCCGATGGCAAACATCACTGTGGATTAC
MR: GGACTAGTTGATCCACCACCAGACC
CBM_F: GGACTAGTGTATCAGGTAACCTTAAGGTGGAG
CBM_R: CTCGAGAACCCGGGGACCAGGTTCCTTTCCCCAC
XmaI_GG_LAPC33S: GGCCCGGGGGCTATCTACCAGCAAAACTATTGATATGG
XmaI_LAP-TG_F1: TCCCCGGGGGCACCATCACC
Fu:EK_OL_R1: CTTATCGTCGTCATCACTGCTTTGCAAATGCTGTGC
Fu:EK _OL_F2: GATGACGACGATAAGGCTCTTGACACTAATTATTGTTTTAGC
XhoI_LAP_TG_R2: GGCTCGAGTCAGCTACATTTACAGGAG
